# Carbonaceous Nanoparticle Air Pollution: Toxicity and Detection in Biological Samples

**DOI:** 10.3390/nano12223948

**Published:** 2022-11-09

**Authors:** Imran Aslam, Maarten B. J. Roeffaers

**Affiliations:** Centre for Membrane Separations, Adsorption, Catalysis, and Spectroscopy for Sustainable Solutions, Department of Microbial and Molecular Systems, KU Leuven Celestijnenlaan 200F, 3001 Leuven, Belgium

**Keywords:** carbonaceous nanoparticles, carbon black, black carbon, brown carbon, toxicity, optical detection

## Abstract

Among the different air pollutants, particulate matter (PM) is of great concern due to its abundant presence in the atmosphere, which results in adverse effects on the environment and human health. The different components of PM can be classified based on their physicochemical properties. Carbonaceous particles (CPs) constitute a major fraction of ultrafine PM and have the most harmful effects. Herein, we present a detailed overview of the main components of CPs, e.g., carbon black (CB), black carbon (BC), and brown carbon (BrC), from natural and anthropogenic sources. The emission sources and the adverse effects of CPs on the environment and human health are discussed. Particularly, we provide a detailed overview of the reported toxic effects of CPs in the human body, such as respiratory effects, cardiovascular effects, neurodegenerative effects, carcinogenic effects, etc. In addition, we also discuss the challenges faced by and limitations of the available analytical techniques for the qualitative and quantitative detection of CPs in atmospheric and biological samples. Considering the heterogeneous nature of CPs and biological samples, a detailed overview of different analytical techniques for the detection of CPs in (real-exposure) biological samples is also provided. This review provides useful insights into the classification, toxicity, and detection of CPs in biological samples.

## 1. Introduction

**Figure 1:**
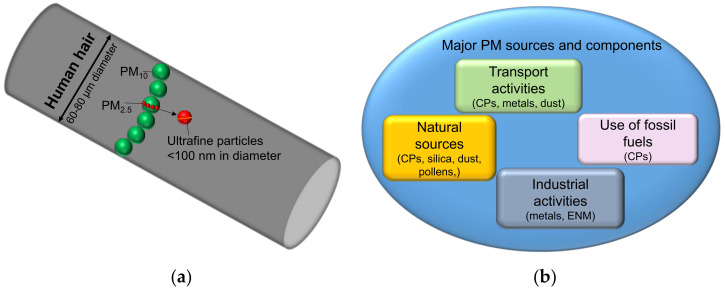
(**a**) Different components of PM based on size: PM_10_, PM_2.5_ and PM_0.1_. With a decrease in the size of PM components, their concentration increases exponentially. (**b**) Major sources of PM pollution and different components from major sources.

Air pollution has been a major concern for many years due to its harmful effects on the environment and public health [1]. Worldwide, outdoor air pollution is estimated to be the cause of approximately four million premature deaths annually, of which about half a million occur in the European Union (EU). An additional four million premature deaths have been attributed to household air pollution in the World Health Organization’s (WHO) report [2,3,4]. Air pollution is a complex mixture of gaseous components and particulates originating mainly from human activities and natural processes [5]. The composition of the pollutants depends on various factors such as their sources, emission rate, and wind conditions [5]. The major gaseous pollutants are nitrogen oxide (NO_2_), ozone (O_3_), and carbon monoxide (CO), whereas common particulates in the atmosphere include various particles of natural and anthropogenic origins [6,7]. Particulate matter (PM), or simply particulates, are extremely small particles and liquid droplets suspended in the atmosphere containing a mixture of different salts, organic chemicals, metals, dust particles, and CPs [8,9]. PM pollutants can be classified based on their sizes into coarse PM (PM_10_; size < 10 µm and >2.5 µm), fine PM (PM_2.5_; size < 2.5 µm), and ultrafine PM (PM_0.1_; size < 100 nm) (Figure 1a). The particulates in PM can either be directly emitted into the atmosphere, i.e., primary PM, or can be formed in the atmosphere from gaseous precursors—the secondary PM [9,10]. Different emission sources from anthropogenic activities and natural processes contribute to the release of the primary components of PM in the environment (Figure 1b) [9]. The major anthropogenic emission sources of PM in the atmosphere are industrial activities, transportation, and the burning of fossil fuel [11,12]. Industrial activities are a major contributor to the presence of metals and engineered nanomaterials (ENMs) in the environment, resulting in elevated levels of metal pollution near sites with industrial activity [13,14,15]. Transportation is also a major source of CPs, dust, and metals in the atmosphere through exhaust and non-exhaust activities. For example, exhaust gases from the incomplete combustion of fossil fuels emit CPs into the atmosphere. Whereas non-exhaust activities such as the erosion of roads, brakes, and tires are major sources of dust and metals in the atmosphere [16,17]. People living near major highways with heavy traffic are more prone to the adverse effects of CPs [18]. The burning of fossil fuel for heating is another major contributor to CP emissions in the environment [19]. Some examples of the major natural CP sources include volcanic eruptions and forest fires [20]. Secondary particles mostly form in the atmosphere through chemical reactions of gaseous pollutants such as the transformation of nitrogen oxides and sulfur oxides [10]. 

Despite the considerable improvements in the atmospheric concentrations of PM recently, the environmental and health effects of PM are still manifold [9,21]. The environmental effects of PM depend on the chemical composition and include global warming, damage to crops and forests, and contribution to acidic rain [22]. The impact of PM on public health is very complex due to their variability in size, morphology, and composition [23]. A few well-known effects include, for example, the worsening of many respiratory diseases such as asthma and chronic obstructive pulmonary disease (COPD) caused by short-term exposure to PM_10_ [24], while exposure to PM_2.5_ has been associated with premature death through existing pulmonary diseases and reduced lung function in children [25]. In addition, exposure to PM_2.5_ results in cardiovascular morbidity and mortality through the deterioration of cardiac function [26]. Epidemiological studies indicate that the carcinogenic effects of PM_2.5_ result in increased mortality, for example, due to lung cancer [27,28]. PM_0.1_ is present in the atmosphere in large concentrations and generally enters the body through inhalation and can translocate to different organs through the bloodstream [29]. PM_0.1_ causes pulmonary inflammation and can be retained in the lungs for a long time. In addition, it is also transported through the olfactory nerve into the brain and hence results in autonomic dysfunction [30,31]. PM_0.1_ exposure is also associated with diabetes, cancer, and low birth weight [32,33].

Since PM is a complex mixture of components with different chemistry, variable sizes, and originating from different sources, it is a complicated task to link the adverse health effects of PM to a specific constituent. Specifically, the fine and ultrafine constituents of PM are more toxic compared to others [23,34,35]. CPs constitute a major fraction of PM_2.5_ and PM_0.1_, and their abundant presence in the atmosphere makes them among the most harmful fractions of ambient PM [23,36,37,38]. Although a few review articles provide insights into the classification of CPs and their detection in environmental samples, this comprehensive review provides useful insights not only into the classification of different CPs but also their toxicity and detection in biological samples. 

## 2. Carbonaceous Particle (CP) Air Pollution

CPs represent a diverse group of materials divided mainly into two major categories: elemental carbon (EC) and organic carbon (OC) [39,40]. EC consists of black carbon (BC), which is directly emitted into the atmosphere through the incomplete combustion of fossil fuels, and carbon black (CB), which is manufactured for use in different applications as a pigment [41]. EC also includes engineered carbon nanomaterials, e.g., carbon nanotubes, fullerenes, and graphene [42]. OC is a complex mixture of different (organic) compounds and can be classified into primary and secondary organic compounds [41]. Among the different components of OC, ambient brown carbon (BrC) is a major fraction resulting from the burning of biomass, the degradation of organic matter, and secondary formation processes in the atmosphere [39,43]. 

### 2.1. Classification of Different Components of CPs

**Figure 2:**
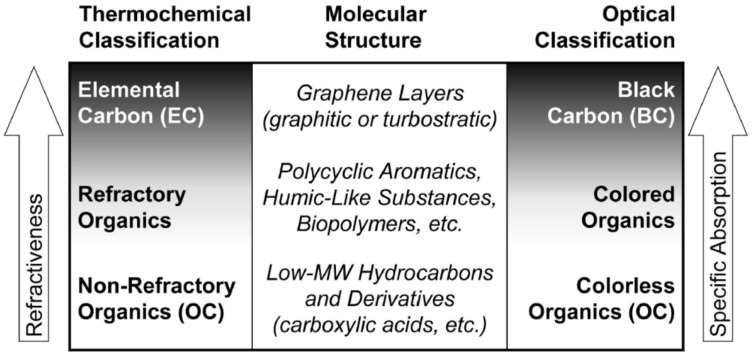
Classification of different components of CPs. Reproduced with permission from Ref. [40]. Copyright © 2002 Springer Nature.

The initial chemical composition of CPs depends mainly on their source. For example, some sources produce almost pure EC, while others produce almost 50% (by mass) OC particles [44]. The internal structure of CPs strictly relates to their optical properties, particularly to those relating to UV–Vis absorption; hence, it is used as a diagnostic tool in different fields such as atmospheric chemistry, solid-state physics, materials science, etc. [45,46,47]. The classification of the different components of CPs based on the most common methods of bulk PM analysis is shown in Figure 2 [39,40]. At the top of this chart, the CB and BC compounds have the strongest optical absorption and lowest volatility. Although CB and BC are inherently complex, their chemical structure and optical properties can be explained by using graphite as an example. Graphite is the most stable (thermodynamically) form of pure EC and is an inert material under atmospheric conditions. Carbon atoms have an sp^2^ orbital hybridization in graphitic structures, which results in a hexagonally symmetric planar arrangement of carbon atoms connected by σ-bonds [48,49,50]. The remaining p-electron is in an orbital perpendicular to the plane of carbon atoms. The p-orbitals overlap sideways to form π-bonds. Due to the infinite planar structure of the graphite sheets, the electrons in the overlapping p-orbitals are delocalized along the hexagonal atomic sheets of carbon [49]. This results in the metal-like properties of graphite, e.g., electrical conductivity and broad-band light absorption. The broad-band light absorption of CPs, particularly CB and BC, also serves as a basis for their optical determination.

Due to the sp^2^ hybridization, the UV–Vis spectra of CB exhibit broad absorption in the UV band between 200 and 250 nm. It also merges with the long wavelength tail of the (σ-σ*) band, located in the far UV end toward 100 nm, which is typical of sp^3^ carbon sites [51]. With the shift of the (π-π*) band’s position toward the visible wavelength, the sp^2^ character increases due to the extension of the sp^2^ hybrid area [48,51]. Hence, this allows us to understand the graphitization process occurring in pyrolysis and combustion systems resulting in the production and CB and BC. Different structural parameters, i.e., the number of stacked graphitic layers and the curvature of aromatic layers, affect the (π-π*) band position [52,53]. The increase in these structural parameters due to the graphitization process causes the shift toward the UV region of the band position, which is opposite to the shift toward the visible region caused by the growth of graphene.

CB and BC exhibit a very complex morphology spanning from the macrostructure to micro- and nano-structures. Even at the nanometer scale, the arrangement of aromatic units of different sizes (>2 rings) in non-stacked and stacked units from two to five stacks can give rise to a different degree of crystallinity and different classes of absorbers [48,54]. This structural complexity leads to changes in the electronic interactions and hence affects the correct interpretation of UV–Vis spectral features of CPs [48].

The bottom of the chart in Figure 1 and Figure 2 shows the volatile organic compounds with their characteristic absorption in the UV range [39]. Between two extremes, the middle of the chart corresponds to the moderately volatile (refractory) compounds with poorly characterized molecular structures. Among these, a few compounds (e.g., HULIS and PAHs) correspond to the colored compounds with optical properties relevant to the BrC particles [47]. Compared to CB and BC, the optical absorption of BrC has strong wavelength dependence in the visible wavelength with the degree of absorption decreasing sharply from the UV to the visible region [39,55]. This is due to the presence of BrC chromophores, and the exact molecular identities of these chromophores are highly variable; however, they are expected to have a high degree of conjugation across the molecular skeleton and high absorption cross-sections. As fluorescent compounds often have the same characteristics, the compounds found in BrC particles can also act as efficient fluorophores [47,56]. In addition, studies have reported that nitroaromatic compounds are major BrC species resulting from the burning of biomass and are also responsible for 50% to 80% of the total light absorption by BrC (at 400 nm) [43,57,58]. Furthermore, aromatic volatile organic compounds, i.e., benzene homologs and derivatives, are also considered important precursors of BrC [57,59]. Specifically, nitrophenols and nitrocatechols are dominant chromophore species (>50% of the concentration) in BrC. In addition, Nitrophenols and nitrocatechols contribute more than 50% of the optical absorption of BrC between 300 and 400 nm [57]. Additionally, anthropogenic volatile organic compounds (e.g., benzene and toluene) and the oxidation of the biomass burning-related products (e.g., pyrocatechol and methylcatechols) can also generate similar BrC chromophores, indicating that these functionalized aromatic compounds play an important role in the optical absorption properties of BrC particles [57]. Furthermore, biogenic materials, their low-temperature oxidation, and polymerization products, e.g., fulvic substances and tannin/lignin compounds, also contribute to the light-absorbing properties of BrC particles [39].

The classification of CPs only based on their optical properties is not sufficient; hence, any potentially available chemical information can also be useful. For example, the H/C molar ratio can be a good indicator of the presence of organic substances in CPs [39]. The H/C molar ratio in BC (soot) is approximately 0.15, and it is well below the values for OC. In highly condensed materials (i.e., PAHs and lignin) it tends to be in the range of 0.5 to 1.5 [39,60]. Atmospheric HULIS have H/C ratios of about 1.4–1.6 [39,61]. Individual CP components are discussed below in detail.

#### 2.1.1. Carbon Black (CB)

CB is an industrially manufactured product consisting of fine black powder obtained through the partial combustion or thermal decomposition of hydrocarbons [42,62]. Based on its manufacturing process, CB can be divided into acetylene black, channel black, furnace black, and thermal black [63]. Approximately ten million tons of CB are produced every year globally, making it one of the top 50 industrially manufactured chemicals [42,64]. Almost 90% of commercially produced CB is used in rubber applications, namely, tire-related automotive uses and other automotive and non-automotive uses of rubber [64,65]. The remaining 10% is used for various applications, e.g., as a black pigment or a conducting agent in plastics, inks, paints, and even in food (E153) [64,65,66,67]. The size of primary CB particles ranges from 15 to 300 nm [42]. There is a huge risk of occupational exposure to CB during different stages of its manufacturing process, production, collection, and handling [42,65]. The risk of occupational exposure to CB is also possible in downstream CB user industries such as the manufacture of rubber, paint, and ink; printing; in the plastics, paper, and ceramics industries; and in carbon electrode production [42,68].

#### 2.1.2. Black Carbon (BC)

BC is a collective term used for different carbonaceous substances ranging from partly charred plant residues to highly graphitized soot particles resulting from incomplete combustion [69]. BC particles are a heterogeneous mixture of different species from various sources consisting of large aromatics and few functional groups [70]. Compared to CB, there is no universally defined chemical definition of BC. However, in a Report to Congress on Black Carbon, the United States (US) Environmental Protection Agency (EPA) defined BC as a “solid form of carbonaceous component of PM that absorbs solar radiation at all wavelengths” [71]. BC mostly originates from the incomplete combustion of biomass and fossil fuels linked to human activities [39,42]. Among these, diesel exhaust engines are a major contributor to the emissions of BC in the environment in the proximity of heavy-traffic areas [72]. In addition, the natural sources of BC emissions are volcanic eruptions and wildfires [73]. The level of global BC emissions from both natural and anthropogenic sources is estimated to be approximately nine million tons per year [42]. The size of BC particles ranges from a few nm to a few hundred nm for atmospheric BC usually sourced from diesel exhaust processes [74]. Atmospheric exposure to BC depends on the daily activities of individuals, whereas persons associated with the transportation sector are prone to higher exposure to BC [72].

#### 2.1.3. Brown Carbon (BrC) 

In contrast to CB and BC, BrC is a fraction of OC that does not absorb all visible light and is characterized by its (light) brown appearance [75]. Recently, BrC attracted the attention of the scientific community due to its strong light-absorbing abilities in the ultraviolet (UV) and visible (Vis) regions and for playing a role in climate change [76]. BrC is chemically very complex as it originates from the incomplete combustion of various materials but can also stem from non-combustion processes either through primary or secondary sources [75]. The major primary BrC sources include low-temperature biomass and coal burning through human activities, biogenic emissions from plant residue, and humic matter [77,78]. Secondary BrC is produced through atmospheric transformation reactions [79]. Compounds of high molecular weight and a light-absorbing nature can result from atmospheric multiphase transformations in the presence of gas-phase or cloud micro-droplets [80,81,82]. BrC is a dynamic mixture of organic compounds and very little information is available about the relationship between its chemical composition and emission sources. Atmospheric humic-like substances have been considered major components of BrC in addition to polycyclic aromatics and biopolymers such as lignin [39,43,61]. Atmospheric BrC can also be classified based on its soluble and insoluble fractions both in water and methanol [83,84]. The global level of BrC emissions is estimated to be approximately seven million tons [75,85]. Although the role of BrC in climate change is already known, the adverse health effects of BrC are also receiving more attention from scientists [86,87]. The characteristics of CB, BC, and BrC are provided in Table 1.

## 3. Environmental Impact of CPs

Carbonaceous aerosols have a significant impact on the weather and climate through the absorption and scattering of sunlight [88]. When suspended in the atmosphere, BC contributes to the warming of the atmosphere by absorbing solar radiation at all wavelengths from UV–Vis to nIR and converting it into heat [89]. BC is the second most important chemical contributing to climate change after carbon dioxide [90]. The BC emitted through human activities and natural sources can also be deposited on the ice and snow, and the consequent decreased albedo effect results in an increased temperature and hence the melting the snow [91]. This also results in the enhanced melting of the ice caps in the arctic and other glaciated regions [92]. In addition, BC also has consequences on the health of the global ecosystem by changing rainfall patterns through its increased absorption of sunlight and by changing the number of liquid cloud droplets [93,94]. This can in turn affect both ecosystems and human living conditions, for example, by affecting agricultural productivity [93,95]. BC can also deposit on the leaves of trees, which consequently increases their temperature [96]. In the past few years, BrC has also received the attention of scientists, particularly in atmospheric research, due to its ability to strongly absorb light in the UV–blue region and hence contribute to climate change [97]. In contrast to BC, the light absorption efficiency of BrC is highly variable as it depends on the chemical composition and source [98]. BrC from the burning of biomass can cause strong circum-arctic warming and indirectly affect the ecosystem and the economy [99,100].

## 4. Health Effects of CPs 

The toxicity of CPs is known to strongly depend on various factors such as their physical characteristics (size, shape, etc.) and chemical composition [101]. Environmental and occupational exposure to ambient CPs is associated with a multitude of diseases as well as a higher mortality rate [102,103,104,105]. CPs from the burning of biomass cause toxicity through the stimulation of oxidative stress, inflammation, and genotoxicity through localized or systemic toxicity [106]. Being a major fraction of ultrafine PM, very fine CPs are highly toxic due to their translocation to different organs via the bloodstream, and a large surface area can result in an enhanced inflammatory response [29]. The toxic effects of CPs on human health are discussed in detail in this section.

### 4.1. Pulmonary/Respiratory Effects

The lungs are the prime organs exposed to atmospheric CPs after inhalation [107]. The deposition of CPs in the lungs depends on the particle size, whereas the composition of the particles determines their toxicity [108]. CB particles consist mainly of EC whereas BC and BrC also contain organic (polyaromatic) components with chemical toxicity towards the lungs [39,42]. Exposure to CPs can cause two pulmonary diseases: COPD and asthma [109,110,111]. The pathophysiology of COPD and asthma includes inflammation of the airways, tissue remodeling and fibrosis, mucociliary dysfunctions, and structural changes [107,112]. Exposure to CPs can also cause alterations in the lungs through the interruption of different lung functions [107]. For example, the inflammation of the airways after the inhalation of CPs causes serious damage to lung function, as diesel exhaust particles (DEPs) alter the production of cytokines [113]. Furthermore, tissue remodeling and fibrosis are observed after inflammatory conditions leading to the accumulation of collagen fibers [107]. Susceptible individuals that already have COPD or asthma are more prone to CP-mediated oxidative damage. In addition, through the generation of reactive oxygen species (ROS), ultrafine CPs cause adverse effects in susceptible individuals with COPD or asthma [114]. The effects of CPs on various biochemical and molecular mediators can cause respiratory dysfunction. In vivo studies have shown the dose-dependent toxicity of synergized CB and Cd resulting in the inflammation of lungs [115]. In an inhalation study, rats were exposed to CB at 7 mg/m^3^ and 50 mg/m^3^ for 6 h per day and 5 days per week. After 13 weeks, inflammation and oxidative stress were observed in the bronchioalveolar lavage fluid from rats [116,117].

### 4.2. Cardiovascular Effects

Exposure to CPs from the atmosphere also affects cardiovascular functions, and several direct or indirect pathways explain the link between CPs and cardiovascular effects [118,119,120]. Through direct pathways, fine, and specifically ultrafine CPs, can translocate from the lungs into the bloodstream and hence target remote organs leading to potential cumulative toxicity [119]. Exposure to ultrafine particles causes cardiac depression effects leading to the deterioration of cardiac function [121]. Indirect pathways are mediated by pulmonary oxidative stress and the inflammatory response, as well as interaction with the autonomic nervous system through specific lung receptors [122]. After the deposition of particles in the lungs, they trigger an inflammation-related cascade resulting in an increased circulating level of pro-inflammatory cytokines, thereby contributing to the risks of atherosclerosis progression [26,123]. The dose-dependent exposure of rats to CB enhanced their cardiovascular risk by inducing hyperhomocysteinemia and platelet hyperactivity [124]. Furthermore, the ROS-dependent mechanism also involves the pro-inflammatory pathway triggered by CPs, which is linked to vascular dysfunction, cardiac arrhythmias, and myocardial infarction [125,126]. In addition, CPs can also stimulate the autonomic nervous system, resulting in impaired autonomic balance and a hyper-activated sympathetic tone that is related to increased cardiovascular risk [127]. Exposure to CPs is also linked with myocardial infarction and accelerated cardiovascular changes [128,129,130].

### 4.3. Reproductive and Developmental Toxicity

Maternal exposure to CPs can result in developmental toxicity and affect three major organ systems in offspring, namely, the central nervous system, the male reproductive system, and the immune system [131]. The dose-dependent maternal exposure to CPs (Printex 90) was linked with changes in the histology of different cell populations in the central nervous system (CNS) and the altered open-field behavior of the offspring in a murine model [132]. Maternal exposure to CPs resulted in the enlargement of lysosomal granules in brain perivascular macrophages (PVMs), as well as increased glial fibrillary acidic protein (GFAP) expression levels in astrocytes, indicating reactive astrogliosis in six-week-old offspring [132,133,134]. The observed changes in the CNS can cause early brain aging, and the offspring have an increased susceptibility to age-related brain disorders [132]. Additionally, the exposure to CPs, particularly CB particles, during gestation has effects on sperm counts and causes structural testicular changes in male offspring [135,136]. The exposure of mice to CB for 10 weeks decreased their daily sperm count and testosterone levels [131]. Maternal exposure to diesel exhaust and tobacco smoke particles can cause allergic immune responses in offspring [137]. Prenatal exposure to CPs can cause immune system impairment, resulting in the frequent onset of allergies during childhood [138]. Maternal exposure to CPs also affects the fetus, resulting in low birth weight [139].

### 4.4. Neurotoxic Effects

Epidemiological research along with in vivo and in vitro studies are further clarifying that exposure to fine particulates damages the nervous system and the brain [31]. Ambient CPs have toxic effects on the brain after potentially translocating through the olfactory tract/olfactory nerve, gastro-intestinal tract/vagus nerve, or blood–brain barrier [140]. The different potential pathways damaging the brain and CNS include direct toxic effects, neuroinflammation, and oxidative stress [141,142]. Ultrafine CPs can have a direct toxic effect through their deposition on the olfactory mucosa of the respiratory tract and subsequent translocation to the brain through the olfactory nerve [31]. Chronic exposure to particles from diesel exhaust can induce oxidative stress, neuroinflammation, and impaired neurogenesis in different brain regions, subsequently leading to brain cell death [143,144]. The dose-dependent exposure of mice to ultrafine soot-iron particles through inhalation was associated with indicators of neural inflammation [145]. Long-term exposure to fine particulates during adulthood accelerates the effects of aging in the brain, thereby increasing the risks of developing dementia or neurodegenerative diseases such as Alzheimer’s disease or Parkinson’s disease [146,147]. Long-term exposure to traffic exhaust CPs is associated with ischemic stroke, resulting in elevated stroke incidences [148]. 

### 4.5. Genotoxic and Carcinogenic Effects

The carcinogenic effects of CPs in humans are either caused at the exposure site such as the respiratory tract or at a distal location after the translocation of particles through the bloodstream [107,149]. Occupational exposure to PAHs, accounting for a significant fraction of BrC and BC, is linked to respiratory, urinary tract, and prostate cancers [150,151]. The regular inhalation of DEPs and CB particles results in pulmonary carcinogenic effects [152,153]. Exposure to CPs, particularly soot, can cause DNA mutations, while higher concentrations of PAHs are responsible for genotoxic effects by damaging DNA [154,155]. Exposure to CPs causes DNA modifications due to DNA adduct formation after the reaction of PAHs with DNA molecules [156,157]. CPs—particularly wood smoke particles—cause oxidative stress, which is associated with a failure of the DNA repair mechanism [158]. Exposure to fine and ultrafine particulates causes DNA methylation changes at the molecular level resulting in changing the expression profiles of genes such that they cause cancer [159]. Soot particles from the 1991 oil fires in Kuwait have shown that a dose-dependent increase results in the induction of genetic effects under in vitro conditions [107]. PAHs also cause epigenetic effects through histone modification, whereas CB can potentiate single- and double-stranded DNA breaks and hence cause genotoxic effects [160,161]. The presence of metal ions in CPs brings about chemical toxicity through the enhanced ROS formation capacity of CPs, resulting in genotoxic effects [162,163]. In addition, BrC particles attract persistent organic pollutants (POPs) and act as carriers for carcinogenic materials such as benzo[a]pyrene (Bap) [75,76,164].

### 4.6. Dermal Toxicity

**Figure 3:**
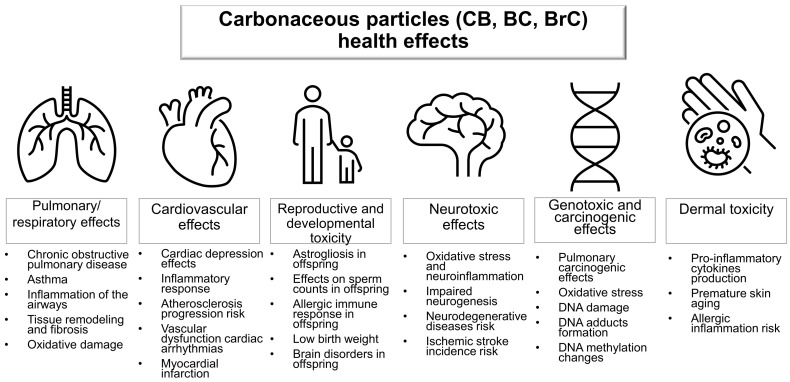
Schematic showing the toxic effects of exposure to CPs in humans.

The skin is the second most important route—after the respiratory tract—for the interaction and penetration of nanoparticle pollutants in the body [165]. Therefore, epidermal cells, like cells in other organs, are exposed to carbonaceous pollutants from the atmosphere, resulting in the production of pro-inflammatory cytokines by human dermal keratinocytes [166,167]. Diesel exhaust CPs and those from cigarette smoke are responsible for damaging effects on skin tissues and premature skin aging [167,168]. For example, DEPs are responsible for the development of allergic and non-allergic skin inflammation after the generation of ROS through redox reactions [169]. Organic extracts from CPs caused dose-dependent cytotoxicity in murine epidermal cells in model studies [170]. Although CPs have cytotoxic effects on the skin, no study was found regarding the toxic effects of CPs in other organs after full penetration through the skin. The toxic effects of CPs on human health are shown in the schematic in Figure 3.

## 5. Characterization Techniques for Carbonaceous Particles (CPs)

Adequate sampling and qualitative and quantitative characterization are essential to evaluate the adverse effects of CPs on the environment and humans. However, CPs’ identification is not a simple task, as they are a heterogeneous group of particulates with a diverse chemical and structural nature and large variability in size [42,47]. In addition, various sampling conditions, such as those presented by polluted air or biological samples, pose many challenges for the qualitative and quantitative identification of CPs [39,171]. For example, elevated background signals from biological settings such as fluids or tissue samples are very intense compared to signals from CPs [171]. Due to the variability in different sampling conditions, it is difficult to develop generally applicable techniques for CPs’ identification [171,172]. The generally used techniques for the characterization of CPs in atmospheric and biological samples are discussed in this section together with their advantages and limitations.

### 5.1. Characterization Techniques for Atmospheric CPs

The identification and quantification of CPs in atmospheric samples is not straightforward and the currently available techniques cannot accurately perform the sensitive detection of the different constituents of PM [37,38,173]. The detection of CPs in atmospheric samples is generally performed using light absorption and thermal radiation techniques; however, these two classes of techniques do not necessarily give similar results [40,94,174,175]. Light absorption techniques correlate the light absorption or attenuation from the samples to the mass (per volume) of the absorbing material using a light absorption coefficient and assuming that the absorbed or attenuated light is proportional to the mass of the CPs [55,176]. Using light absorption techniques, the bulk analysis of CPs is performed using a filter-based approach in which a gas stream (polluted air) is passed through a filter to concentrate the samples. Aethalometers use a filter-based technique to measure the light absorption of CPs and can estimate the contribution from different CPs based on wavelength-dependent light absorption measurements [176,177]. For example, emissions from the burning of fossil fuel absorb light dominantly from the Vis–nIR region, indicating the presence of BC, whereas emissions from the burning of biomass have more BrC, and hence an enhanced absorption in the UV and blue wavelengths [178,179]. In addition, based on the attenuation coefficient, the concentration of the CPs can be estimated [180]. Although this technique can identify and quantify CPs in environmental samples, there are several limitations to this technique such as: (i) the shadowing effect, (ii) the scattering of the light beam at the filter fibers, (iii) and a false response from non-CPs, which can lead to the false estimation of these measurements [176,181]. 

Thermal radiation techniques work on the principle of measuring the mass concentrations of CPs based on heating the light-absorbing carbonaceous aerosols followed by the analysis of emitted radiation [182]. Laser-induced incandescence (LII) is a powerful thermal radiation technique in which the emission signal from the particles is generated by the absorption of high-intensity laser radiation, and the thermal emission from the heated particles is detected in the selected detection window [175,183]. Depending on the laser’s wavelength, LII can identify different fractions of CP in gaseous samples [182]. It can also quantify the mass concentrations (based on the mass per volume) of CPs, as well as measure the particle size based on the temporal decay of the LII signal [184]. It is used extensively for environmental applications such as ambient air quality or source monitoring [182]. There are several limitations of this technique; for example: (i) the instrument response depends on the type of CP, (ii) proper calibration is required to convert the intensity of the thermal radiation to the CP mass, and (iii) no established reference materials are available for calibration [174,181]. 

Furthermore, the combination of LII and light scattering from particles has also been employed to identify CP, as well as to measure their mass concentrations and particle size. This method is used in Single Particle Soot Photometers (SP2) for single-particle analysis [185,186]. In SP2, LII measures CPs such as BC, whereas single-particle light scattering is employed to measure the particle size and mass concentrations [186]. This instrument can also measure the content of CPs without interference from semi-volatile materials or mineral dust particles [175,186]. As SP2 works based on LII and light scattering, the limitations of this method in addition to LII’s drawbacks include (i) the requirement for prior information about the refractive index and shape of the particles, and (ii) the necessity of calibrating the instrument for the accurate determination of the mass concentrations and particle size [175]. 

Other common optical techniques used for atmospheric CP measurements include cavity ring-down spectroscopy (CRDS) and multi-angle absorption photometer (MAAP) [187,188,189]. In addition, high-performance liquid chromatography (HPLC), as well as mass spectrometry (MS), are used for the characterization of chromophores in BrC samples from different emission sources [58,190]. The above-mentioned techniques are briefly discussed for atmospheric samples; however, the detailed discussion of atmospheric CP characterization exceeds the scope of this work. There is an evident need for the characterization of CPs in biological samples in order to gain insights into the toxicity of CPs towards humans. 

### 5.2. Characterization Techniques for CPs in Biological Samples

Currently, the epidemiological research studying the toxic effects of CPs in biological samples is hampered by the scarcity of analytical tools that function in biological samples [171]. Most of the available techniques for the detection of CPs cannot be used because of their limited biocompatibility and the interference of background signals from biological samples [171]. Indirect measurements can make use of radiolabeled CPs to study the toxic effects of CPs in model organisms after deliberate exposure. Further mass spectrometry, electron microscopy, and optical techniques can—to some extent—also be employed for studying CPs from natural exposure. 

#### 5.2.1. Isotope Tracing/Radiolabeling Detection of CPs

Isotope tracing is a useful technique for tracking carbonaceous nanomaterials in different biological systems [191]. This approach has been used to trace CPs in lab animals as well as in clinical studies [191,192,193]. In animal studies on mice, the translocation of ultrafine carbon particles (elemental ^13^C) from the respiratory tract, via the bloodstream, to the liver was observed [191,192]. Human respiratory detection and clearance studies use scintigraphy methods such as CPs labeled with gamma-ray-emitting radioisotopes, and gamma-ray emission can be used for the localization and quantification of CPs in the lungs as well as the clearance and translocation of these particles [194,195]. 

Among the different clinically employed radiolabeling techniques, the most predominant is the “Technegas approach”, which uses ultrafine CP suspensions labeled with Technetium-99m (^99m^Tc) [196]. The preparation of Technegas is carried out in a special machine at 2550 °C in an atmosphere of 100% argon gas and carbon, resulting in a thin layer of technetium encapsulating the carbon nanoparticles with a typical size of 30 to 60 nm [196,197]. The limitations of the employed isotopes in the Technegas technique are as follows: (i) ^99m^Tc has a short physical half-life (~6 h); (ii) ^99m^Tc leaches from the CPs, which limits this technique’s use for clearance studies; and (iii) the hygroscopic properties of the particles as well as the presence of free pertechnetate can cause chemical instability of the generated particles [193,196,198]. Therefore, many modifications to this technique are used such as labeling with an indium isotope (^111^In) or ^68^GaCl_3_, which can extend the investigation time from a few hours to a few days [198,199,200]. Although these labeling methods offer sensitive localization and quantification of carbon nanoparticles in human studies, they have limitations, such as exposing the subject to radiation; the use of labeled carbon nanoparticles, which can modify the properties of the nanoparticles; and the limited post-administration follow-up, as radionuclides decay after few days [171,196]. 

#### 5.2.2. Mass Spectrometry Detection of CPs

Laser desorption/ionization mass spectrometry (LDI-MS) is widely used for the characterization of CPs in environmental samples, and has been recently employed for the identification of CPs in biological samples such as cells and those in animal studies [86,201]. LDI-MS works on the principle of the ionization of the analyte using a laser beam, thereby removing the molecules from the surface of the material and ionizing them [202]. Afterwards, the ionized molecules are characterized based on their mass-to-charge ratio (m/z) in the analyzer through the time-of-flight (TOF) measurements [203,204]. Lin et al. used LDI-MS to study the biodistribution of soot particles in mice after their inhalation of PM_2.5_ [201]. The samples with CPs were ionized under laser excitation in a vacuum atmosphere resulting in the formation of carbon cluster structures and characteristics peaks (C_n_^−^) with repeated mass units [201]. The identification and quantification in biological samples were performed using murine lung samples and consistent anionic carbon cluster peaks were observed, making this technique feasible for measurements in biological samples [201]. The use of mass spectroscopy to identify and quantify CPs (soot) in biological samples is very promising; however, there are limitations of this approach, for instance: (i) the removal of the impurities can make sample preparation very complicated, and (ii) mostly biological samples contain carbon; hence, mass spectrometry can result in the detection of carbon cluster peaks from biological samples instead of soot particles [205,206].

#### 5.2.3. Electron Microscopy Detection of CPs

**Figure 4:**
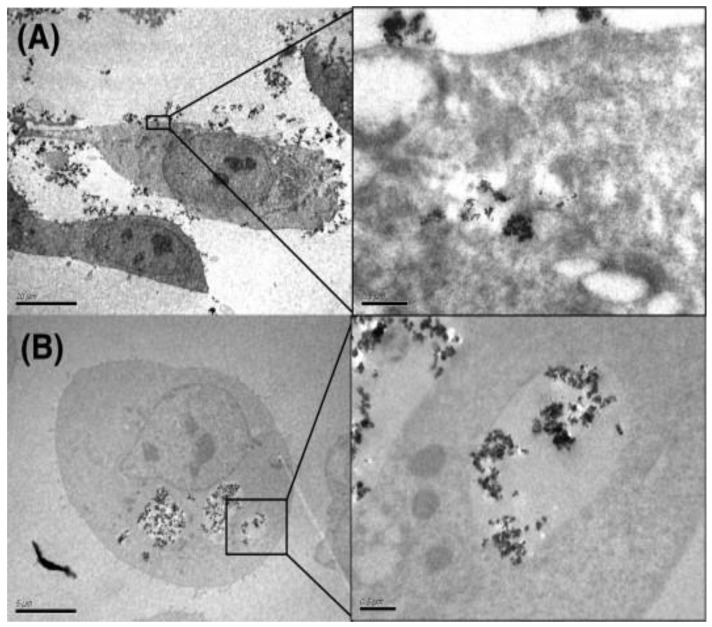
TEM images of 16HBE cells incubated with CB micro- and nanoparticles (MNPs). (**A**) 16HBE cell with CB13 MNPs in a vesicle (scale bars: left (10 µm); right (0.5 µm)) (**B**) 16HBE cell containing CB21 MNPs in a vesicle (scale bars: left (5 µm); right (0.5 µm)). Reproduced with permission from Ref. [211]. Copyright © 2011 Elsevier.

Electron microscopy (EM) is a conventional technique that can provide good spatial resolution to analyze the cellular uptake of nanoparticles [207,208,209]. By using EM, CPs are characterized based on their typically black aggregates in the cells or tissue samples [210,211]. Scanning electron microscopy (SEM) scans the surface of a sample using a focused electron beam and can provide images of the sample in 3D with a very high resolution (3–20 nm) [212,213]. The detailed visualization and internalization of the nanoparticles by a cellular organism can be performed by transmission electron microscopy (TEM), in which a beam of electrons is transmitted through a thin specimen [213]. TEM analysis also provides information about different parameters of the nano-particulates such as their size distribution, shape, and aggregation [213]. Owing to its sub-nanometer resolution, it can reveal the fine relationships between the nano-particulates and the cellular/tissue components [210,213]. For example, Jiang et al. visualized the cellular uptake of CB in the cytoplasm of the BEAS-2 cells. Komatsu et al. also observed CB and DEPs in the form of randomly dispersed aggregates in the cytoplasm of TM3 cells, whereas Zhang et al. found the particles trapped inside the alveolar macrophages from measurements of the lung sections of mice exposed to CB [209,210,214]. In addition, Belade et al. observed the distribution of CB NPs in MRC-5 and 16HBE cells using TEM and found aggregates in the cytosol and cytoplasmic vesicles (Figure 4) [211].

TEM can provide a very high resolution; however, some of the limitations of this technique are as follows. (i) CPs can only be identified based on their dark appearance in a bio-context, hence, it is difficult to discriminate different CPs. (ii) Biological samples need to be prepared carefully by skilled persons and sliced into very thin sections for analysis using TEM. (iii) The small field of view of TEM can hamper the imaging of large biological specimens such as entire organisms. (iv) Finally, TEM can only provide a static snapshot of the sample; thus, the investigation of dynamic cellular processes is limited [215,216]. Although conventional EM is widely used in NP research in biological systems, it cannot be used as the only tool for the qualitative or quantitative evaluation of the cellular uptake of NPs [217]. Hence, to obtain more abundant and accurate information, it is mostly combined with other methods such as optical microscopy techniques, electron energy loss spectroscopy (EELS), or energy dispersive X-ray (EDX) [218]. Another drawback of EM is that it involves the use of a very expensive instrument compared to most optical microscopes [213].

#### 5.2.4. Optical Techniques for the Detection of CPs

Optical techniques can offer the capabilities of the non-destructive and label-free detection of CPs in their biological context. These techniques work based on different phenomena, including the optical absorption or scattering of light, molecular vibrational fingerprinting, or exploiting the non-linear optical behavior of nanoparticles.

- Absorption- and scattering-based microscopy

Absorption-based microscopy is one of the most straightforward forms of optical microscopy techniques and can be implemented in a bright-field microscopy system [219,220]. It works on the principle of the transmission of light through the sample and the generation of contrast based on the changes in the absorption of light in the denser regions of the sample [219]. Therefore, CB/BC NPs will appear darker compared to BrC NPs against the transparent biological media in the bright-field images [39,219]. In comparison, scattering-based microscopy works on the principle of the detection of Rayleigh scattering from the specimen, in which contrast between different materials is generated based on the scattering intensity [219,221]. The scattering intensity from the particles in a biological context depends not only on their size but also on their refractive index, the refractive index of the surroundings, and the optical configuration of the setup in which the scattered light is detected [222]. Scattering-based microscopy images can be obtained using a conventional dark-field microscope [219]. Hence, both of these optical techniques can be applied for the label-free observation of CPs based on their light absorption or scattering in biological fluids, as well as their uptake by cells or tissues [171].

You et al. observed the nanoparticulate CB in the lung CD11c^+^ cells from mice deliberately exposed to CB using bright-field and dark-field microscopy [223]. In addition, Modrzynska et al. observed the black aggregates in the liver sections of mice exposed to CB NPs using bright-field microscopy [224]. Absorption- and scattering-based microscopy are simple yet effective approaches to the visualization of CPs in biological samples, but they are limited by: (i) low resolution; hence, the detection of very small CPs below ~200 nm is challenging and only large aggregates can be detected; (ii) visualization at a low concentration is difficult; and (iii) the identification of different types of CPs is not possible [171,225].

- Raman spectroscopy

**Figure 5:**
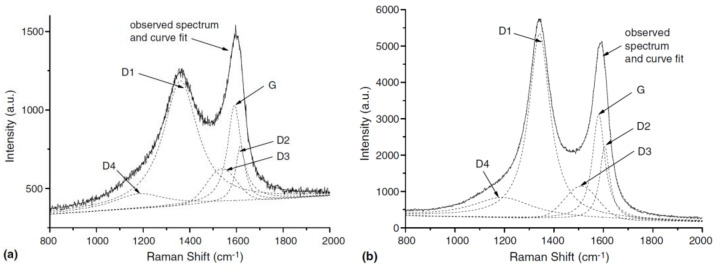
Peak fitting of first-order Raman spectra obtained using 514 nm laser. (**a**) Diesel soot; (**b**) Printex XE2. Reproduced with permission from Ref. [232]. Copyright © 2005 Elsevier.

Raman spectroscopy is a valuable technique for the label-free quantitative and qualitative characterization of CPs in biological samples [226,227]. It works on the principle of detecting inelastically scattered light to study the vibrational fingerprinting of different materials for their characterization [228]. Raman spectroscopy is used to obtain detailed information on different types of carbonaceous materials and their degrees of structural disorder [229]. Different types of CPs are distinguished based on their degree of graphitization using Raman spectroscopy [226,230,231]. The features of the first-order Raman spectra of carbonaceous materials are in the region from 1200 cm^−1^ to 1700 cm^−1^, whereas second-order features are around 2700 cm^−1^ and can range up to 3500 cm^−1^ [226,232]. The first-order Raman transitions provide information about carbon material with a long-range order (graphitic carbon) or without a long-range order (amorphous carbon) [226]. Amorphous carbon is mostly an unknown mixture of sp^2^- and sp^3^- bonded carbon [226]. The most important Raman features observed for carbonaceous materials are around 1580 cm^−1^, representing an ideal graphitic lattice (G-band), and at ~1350 cm^−1^, representing a disordered graphitic lattice from graphene layer edges (D1-band) (Figure 5) [232,233]. Some smaller features are also observed at ~1620 cm^−1^ (D2-band), ~1500 cm^−1^ (D3-band), and ~1200 cm^−1^ (D4-band) [232,233]. The D2-, D3-, and D4-bands represent the disordered graphitic lattice at the surface graphene layers, amorphous carbon, and a disordered graphitic lattice due to polyenes or ionic impurities, respectively [233,234,235].

Although Raman spectroscopy is mostly used for the characterization of CPs in environmental samples, few studies have reported the use of Raman spectroscopy for characterizing carbon materials in biological samples [223,236,237,238]. For example, Knief et al. studied the suitability of Raman spectroscopy to determining the toxicity of carbon nanotubes in human epithelial cells (A549) [236]. In addition, the Raman fingerprints of CPs in the lungs of mice exposed to cigarette smoke for four months were observed [223]. Furthermore, Čabanová et al. detected the Raman fingerprints of amorphous carbon in the mucosa and hypertrophic tissue samples of patients with chronic rhinitis, which indicates the potential of Raman spectroscopy to identify CPs in real exposure measurements [237,238]. 

Raman spectroscopy has the potential to characterize CPs based on their vibrational fingerprint; however, the application of Raman spectroscopy in most biological samples is limited by the strong autofluorescence background signal (from PAHs in BrC) [227,228]. In addition, to achieve a good signal-to-noise ratio (SNR), long acquisition times are needed, which greatly slows the associated measurements [227]. Hence, acquiring Raman measurements of CPs in (bio)liquid samples is not a simple task due to the long measurement times. A very weak Raman-scattering signal can be compensated with higher laser power densities, which can be detrimental to biological samples [171,228].

- Photothermal pump–probe microscopy

Pump–probe microscopy is a label-free optical technique used for the characterization of different types of nanoparticles in biological samples [239,240]. The basic idea behind the pump–probe approach is that the pump field excites the sample to a higher energy state or perturbs the electronic states of the materials, whereas the probe field determines the changes in the electronic states [240]. The probe field’s absorption is either transiently enhanced or reduced [240,241]. In transient absorption microscopy, the absorption of the probe beam is transiently altered by the pump field, resulting in short-term changes in the probe field’s intensity [240,242]. Ground-state depletion microscopy is another type of pump–probe microscopy approach, in which the population of the ground state of a molecule is altered and hence results in the reduced absorption of the probe field [243]. The photothermal approach to pump–probe microscopy also uses a pump field to heat the specimen in focus and induce changes in the refractive index of the surrounding medium, and modifications in the probe beam are observed due to the induced heat [244].

Steuwe et al. used photothermal pump–probe microscopy to identify CPs in different biological samples based on the contrast generation due to changes in the probe field intensity [245]. In addition to visualizing CB NPs in an automated fashion in a flow cell, CB NPs were also detected inside the human lung fibroblasts and spiked urine samples. The nonlinear nature of the signal provides 3D sectioning as well as large imaging depths due to the use of longer laser wavelengths. 

This technique successfully detected CPs in different biological samples; however, some of the limitations of this approach are as follows: (i) the non-specific nature of the signal makes it difficult to distinguish different types of CPs; (ii) typically, the pump field can excite several molecular states simultaneously and result in the generation of spurious signals from non-CPs; and (iii) pump–probe microscopy requires the use of two ultrafast light sources mainly comprising a laser and an Optical Parametric Oscillator (OPO), hence making it an expensive approach [239,246]. Pump–probe approaches have also been proposed using cheaper diode laser; however, this can compromise the resolution [247,248].

- Femtosecond-pulsed laser microscopy (non-incandescence-related white light generation) for CP detection

Femtosecond- (fs) pulsed laser microscopy (FPLM) is an innovative approach for the label-free and non-invasive characterization of CPs in fluids, cells, and tissue samples [249]. This approach can be implemented using a multiphoton microscopy setup available in most biomedical research labs [250]. Some of the non-linear optical processes observed using a multiphoton microscope include two-photon-excited autofluorescence (TPAF), second-harmonic generation (SHG), third-harmonic generation (THG), etc. [250,251,252,253]. These non-linear processes work based on the simultaneous absorption of two or more photons by the specimen [250,254]. The multiphoton approach has the advantages of imaging living tissue samples with greater penetration depth and lower radiation damage due to the use of nIR laser wavelengths [250,255]. 

**Figure 6:**
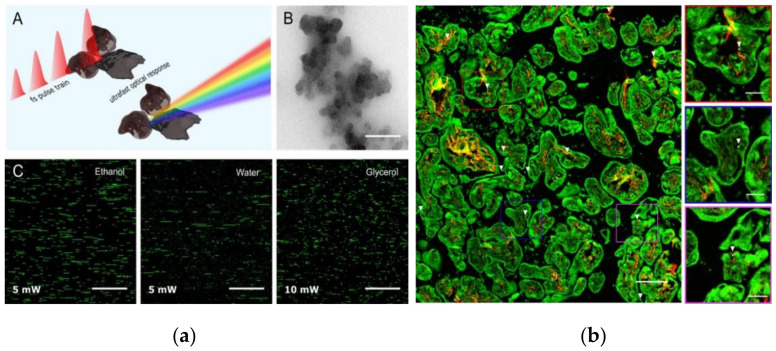
(**a**) The detection method of CB NPs based on WL emission: (**A**) Schematic showing the emission and excitation process. (**B**) TEM image of CB NP aggregates. Scale bar: 300 nm (CB). (**C**) CB detection using the FPLM detection method in different sampling conditions. Reproduced with permission from Ref. [249]. Copyright © 2016, American Chemical Society (ACS). Available from: https://pubs.acs.org/doi/10.1021/acs.nanolett.6b00502 (accessed on 14 April 2022). Further permissions related to the material excerpted should be directed to the ACS. (**b**) Detection of BC particles at the fetal side of human placenta based on WL emission originating from the BC particles under illumination with fs-pulsed laser. Reused from Ref. [258], originally published under Creative Commons Attribution 4.0 International License, Copyright © Authors 2019.

Recently, Bové et al. developed a novel biocompatible approach based on fs-pulsed laser microscopy for the label-free identification of CPs in biological media (Figure 6a) [249]. This approach works on the principle of non-incandescence-related white light (WL) generation from CB NPs when illuminated using a fs-pulsed laser at nIR wavelengths. The light emitted by the CB NPs covers the whole visible spectrum, and based on this spectral information, the emitted light can be detected in different spectral windows compatible with most multiphoton microscopes [256]. By using this approach, BC particles were detected in urine samples, human placenta samples (Figure 6b), and on plant leaves [257,258,259]. Further, the relationship between the WL emission intensity and particle size was also reported in aqueous suspensions [260]. Using FPLM, Bongaerts et al. recently revealed the presence of ambient BC particles in fetal blood and organs [261]. Table 2 provides a detailed overview of the techniques for the detection of CPs.

## 6. Conclusions and Outlook

### 6.1. Conclusions

CPs, being a major fraction of ultrafine PM, are abundant in the atmosphere and originate from different anthropogenic and natural sources. Their abundant presence in the atmosphere not only contributes to environmental impacts such as global warming (by their absorption of solar radiation) but also presents a likely pathway for their inhalation by humans. The inhalation of CPs by humans results in a wide range of adverse effects, ranging from respiratory to carcinogenic effects, as ultrafine CPs can translocate through the bloodstream to different organs. The qualitative and quantitative detection of CPs in atmospheric and biological samples is very important to understand their adverse effects. Different analytical techniques such as laser-induced incandescence and optical absorption are used for CPs’ detection in gaseous samples. However, CPs’ detection in biological samples is very challenging due to their heterogeneous nature and complexity associated with biological samples. The different analytical techniques used for CPs’ detection in model studies in biological samples include radiolabeling detection, electron microscopy, mass spectrometry, etc. Whereas Raman spectroscopy and FPLM are employed for CP detection in models and real-exposure samples.

### 6.2. Challenges and Outlook

Different techniques such as FPLM or Raman spectroscopy are very sensitive for the qualitative and quantitative detection of CPs in real-exposure biological samples; however, these microspectroscopy techniques are very labor-intensive and time-consuming due to the related sample preparation, microscopy measurements, and data analysis. Therefore, to detect CPs in biological samples for clinical applications, there is still a need for the development of robust, sensitive, and time-effective analytical techniques. There is also growing evidence that the harmful effects of PM pollutants are size-dependent, in wherein ultrafine particles are the most harmful [262]. Hence, the size determination of CPs in biological samples can provide additional information about the toxicity of CPs related to their size. The techniques discussed in this review, e.g., FPLM and Raman spectroscopy, cannot detect very small particles (<100 nm) in biological samples; hence, high-resolution techniques are needed to detect particles below 100 nm. 

Although BC and CB constitute a major fraction of CPs, recently, BrC has also received significant attention from scientists. The complexity and heterogeneity of CPs, the variability of their molecular properties, and the nature of their emission sources pose great challenges to their detection and discrimination in biological samples. Hence, there is a need for the development of advanced and sensitive analytical tools for the qualitative and quantitative detection and discrimination of different components of CPs to understand their adverse effects on human health. The currently available approaches will require optimization to not only detect very small particles (<100 nm) but to also discriminate between different CPs for diagnostic purposes. 

In addition to understanding the adverse effects of CPs on human health, their atmospheric concentrations from different anthropogenic sources need to be controlled by better policy making (and implementation) at the regional and national levels. Recently, the WHO released new Global Air Quality Guidelines intending to save millions of lives from the harmful effects of air pollution. Among the different constituents of air pollution, particulate matter (PM) is on the top of the agenda of the WHO guidelines. In addition, the EU key directive (2008/50/EC) on ambient air quality and cleaner air for Europe also stressed reducing air pollution levels to minimize the adverse effects on human health. 

## Figures and Tables

**Table 1:** Characteristics of CB, BC, and BrC.

Characteristics	Carbon Black (CB)	Black Carbon (BC)	Brown Carbon (BrC)
**Sources/Origin**	Large-scale commercial production processes, abrasion of materials containing CB.	Biomass burning, diesel exhaust, forest fires, volcanic eruptions.	Biomass burning, coal burning, forest fires, biogenic sources (dust, humic matter, etc.).
**Production/Emission estimates**	~9.8 million tons per year.	~8.5 million tons per year.	~6.9 million tons per year (carbon), variable.
**Composition/Molecular structure**	Elemental carbon > 97%	Source-dependent, often elemental carbon > 50%	Humic-like substances, PAHs, Biopolymers.
**Primary particle sizes**	~15–300 nm	Smaller than primary CB particles: diesel exhaust around ~15–40 nm.	Various sizes, depending on the molecular structure.
**General morphology (shape, form)**	Aciniform aggregates and agglomerates.	Complex chains, aciniform aggregates, agglomerated spherical particles.	Individual particles, aggregates with PAHs, tarballs.
**Exposure sources**	Environmental and Occupational.	Environmental: anthropogenic and natural sources.	Environmental: anthropogenic and natural sources.
**Environmental/Health effects**	Cytotoxicity, inflammation and oxidative stress, cardiovascular and respiratory diseases.	Global warming, cytotoxicity, inflammation and oxidative stress, cardiovascular and respiratory diseases, carrier for harmful substances.	Global warming, cell apoptosis, carrier for carcinogenic NPs, persistent organic pollutants (POPs).
**Optical properties**	Absorbs light at all wavelengths from UV–Vis-nIR.	Absorbs light at all wavelengths from UV–Vis-nIR.	Absorbs light strongly in the UV region.
**References**	[42,62,63,64,65,67]	[39,42,69,70,71,72,73,74]	[39,43,61,80,81,82,83,84,85,86]

**Table 2:** Techniques for detection of CPs in biological samples.

Detection Techniques	Detection Mechanism	Sample Types	Label-Free Approach	Model Studies	Detection in Real Samples	Limitations	References
**Radiolabeling detection**	Half-life probe	Cells, tissues, clinical studies	No	Possible	Not Possible	Individual gets exposed to ionizing, limited post-administration follow-up, limited to deliberate exposure.	[171,194,195,196,198]
**Mass spectrometry**	Laser desorption/ionization mass to charge ratio	Solid/liquid samples	Yes	Possible	No studies reported	Removal of impurities, complicated sample preparation, interference from the biological samples.	[86,201,203,204,205]
**Electron microscopy**	Using a focused electron beam	Dried samples	Yes	Possible	No studies reported	Complicated sample preparation, small field-of-view, investigation of dynamic cellular processes are not possible.	[207,208,210,211,212,213,215]
**Absorption- and scattering-based microscopy**	Light absorption and scattering	Solid/liquid samples	Yes	Possible	No studies reported	Low resolution, detection of very large aggregates, challenging to detect low concentrations.	[171,219,220,221,222,223,224]
**Raman spectroscopy**	Molecular vibrational fingerprinting	Solid/liquid samples	Yes	Possible	Possible, detected in human nasal mucus and hypertrophic tissues	Strong background signal from cells or tissues, long acquisition times.	[171,226,227,228,229,230,231,232,233,234,235]
**Photothermal pump-probe microscopy**	Contrast generation due to changes in the probe field intensity	Solid/liquid samples	Yes	Possible	No studies reported	Generation of spurious signal from non-CPs, cannot distinguish among different CPs, expensive.	[239,240,241,242,244,245,246,247,248]
**Femtosecond pulsed laser microscopy (non-incandescence related WL generation)**	White light emission, spectral information	Solid/liquid samples	Yes	Possible	Possible, detected in human urine and placenta samples	Possible interference from non-CP components of PM, size determination is challenging, difficult to distinguish among different CPs.	[249,250,251,252,253,255,256,257,258,259,261]

## Data Availability

Not Applicable.
